# Case Report: Hypereosinophilic syndrome vs. patent foramen ovale as etiopathogenetic contributors to stroke

**DOI:** 10.3389/fcvm.2023.1298063

**Published:** 2024-01-12

**Authors:** Xiangjuan Liu, Congcong Sun, Guipeng An, Lili Cao, Xiao Meng

**Affiliations:** ^1^ National Key Laboratory for Innovation and Transformation of Luobing Theory, The Key Laboratory of Cardiovascular Remodeling and Function Research, Chinese Ministry of Education, Chinese National Health Commission and Chinese Academy of Medical Sciences, Department of Cardiology, Qilu Hospital of Shandong University, Jinan, China; ^2^ Department of Neurology, Qilu Hospital, Cheeloo College of Medicine, Shandong University, Jinan, China

**Keywords:** hypereosinophilic syndrome, patent foramen ovale, cerebral infarction, optical coherence tomography, case report

## Abstract

Hypereosinophilic syndrome (HES), characterized by an increased number of eosinophils in tissues and/or blood, presents with heterogeneous clinical manifestations. Studies have shown that HES can affect the nervous system and may be associated with cerebral infarction. Patent foramen ovale (PFO) is the most common congenital intracardiac defect that can cause right-to-left shunting and contribute to the paradoxical embolization of venous emboli, and even lead to stroke. We report the case of a young man who presented with cerebral infarction accompanied by both HES and PFO. The patient underwent thorough evaluation to determine the source of emboli and the potential pathogenesis. In this case, HES was confirmed and glucocorticoid treatment was conducted. Direct imaging using optical coherence tomography (OCT) confirmed that the embolus originated from the PFO. Therefore, we performed PFO occlusion. The patient recovered well, and no new cerebral infarction was observed at 6-month follow-up. Based on the results of our study, we conclude that it is important to consider unusual etiologies of cerebral infarction, particularly in younger patients.

## Introduction

Hypereosinophilic syndrome (HES) is a relatively rare disorder and the pathogenesis is not entirely clear. It affects predominantly males (4-9:1 ratio), and is extremely rare in children. The exact prevalence of HES is still unknown (1). HES is defined as persistent elevation of the peripheral blood eosinophil count (>1.5 × 10^9^/L) associated with evidence of eosinophil-induced end-organ injury and dysfunction (2, 3). HES presents with heterogeneous clinical manifestations, including dermatologic, pulmonary, gastrointestinal, and cardiac lesions (2). Although eosinophilia-associated stroke is a common and severe complication of HES, its pathogenesis remains unclear.

Patent foramen ovale (PFO) is the most common congenital intracardiac defect, which affects approximately 25.0% of the adult population (4). PFO can cause right-to-left shunting and contribute to the paradoximal embolization of venous emboli to reach the left atrium through the PFO, followed by subsequent cerebral embolization and leading to stroke (4). Several studies have reported an association between PFO and stroke in young patients (5).

In this study, we describe a young man who presented with cerebral infarction accompanied by both HES and PFO, and underwent comprehensive evaluation to determine the source of emboli and the potential pathogenesis.

## Case presentation

**Figure 1:**
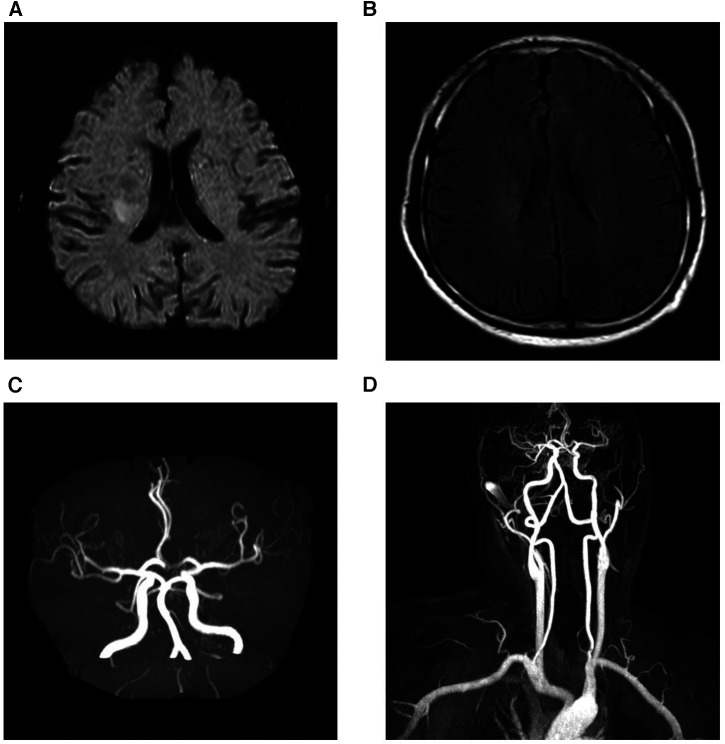
Brain magnetic resonance imaging. Diffusion weighted images (**A**) and fluid-attenuated inversion recovery weighed images (**B**) revealed that acute multiple cerebral infarction was present in bilateral basal ganglia and corona radiate. Magnetic resonance angiography of cranial (**C**) and carotid arteries (**D**) showed no significant stenosis of the cerebral, carotid, or vertebral arteries.

A 43-year-old man presented with a 1-week history of left extremity weakness. The patient was a non-smoker and denied a history of hypertension, hyperlipidemia, diabetes, migraine or cerebrovascular disease. He had no clinical history of parasitic infections or malignant or allergic diseases. On physical examination, the patient was conscious, and cardiac examination revealed a regular rhythm without arrhythmias. However, muscle strength was reduced to grade 3 in his left extremity. Brain magnetic resonance imaging (MRI) revealed multiple acute cerebral infarctions in the bilateral basal ganglia and corona radiata (Figure 1), and magnetic resonance angiography (MRA) showed no significant stenosis of the cerebral, carotid, or vertebral arteries. The patient was diagnosed with cerebral infarction and was admitted to the neurological ward.

During the hospitalization, the patient received dual antiplatelet therapy, including aspirin and clopidogrel, along with atorvastatin treatment. 24-hour Holter monitoring was performed in this patient, revealing sinus rhythm with occasional atrial and ventricular premature beats. No atrial fibrillation was detected. On laboratory investigations, his hemogram showed a significant increase of leukocytosis (17.73 × 10^9^/L) and eosinophilia (9.66 × 10^9^/L), with a high eosinophil ratio of 54.6%. His coagulation profile was normal, except for a slightly elevated D-dimer level (0.8 μg/mL).

**Figure 2:**
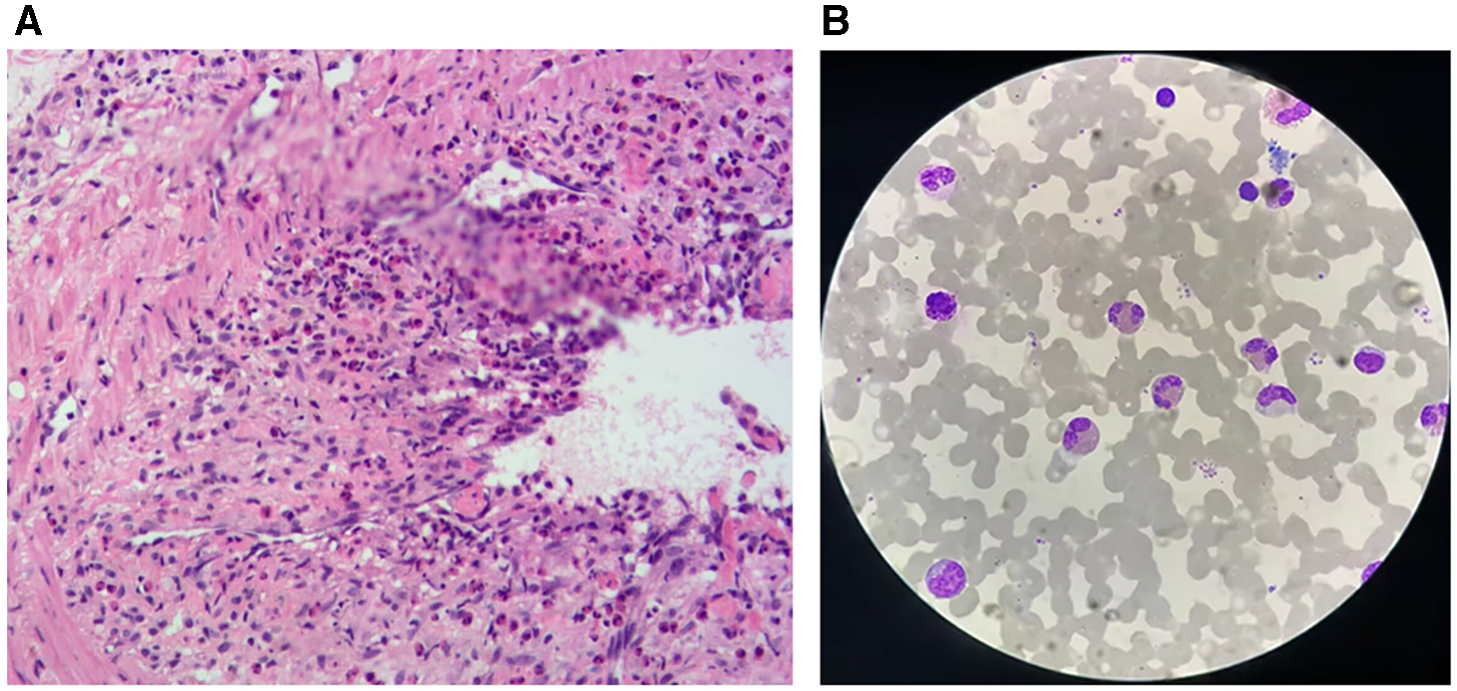
(**A**) Skin biopsy of the right lower limb. (**B**) Bone marrow biopsy.

Screening for JAK-2 protein and F1P1L1/PDGFRa gene mutations yielded negative results. Evaluation of a skin biopsy specimen obtained from the right lower extremity revealed histopathological changes indicative of eosinophilic vasculitis (Figure 2A). A bone marrow biopsy revealed relatively pronounced eosinophilia [eosinophilic granulocytosis (34.0%)] (Figure 2B). Chromosomal analysis was unremarkable. Therefore, the patient was diagnosed with idiopathic hypereosinophilia and received glucocorticoid treatment (methylprednisolone). After 6 days of therapy, the leukocyte count decreased to 8.47 × 10^9^/L with 1.4% eosinophils (0.12 × 10^9^/L).

**Figure 3:**
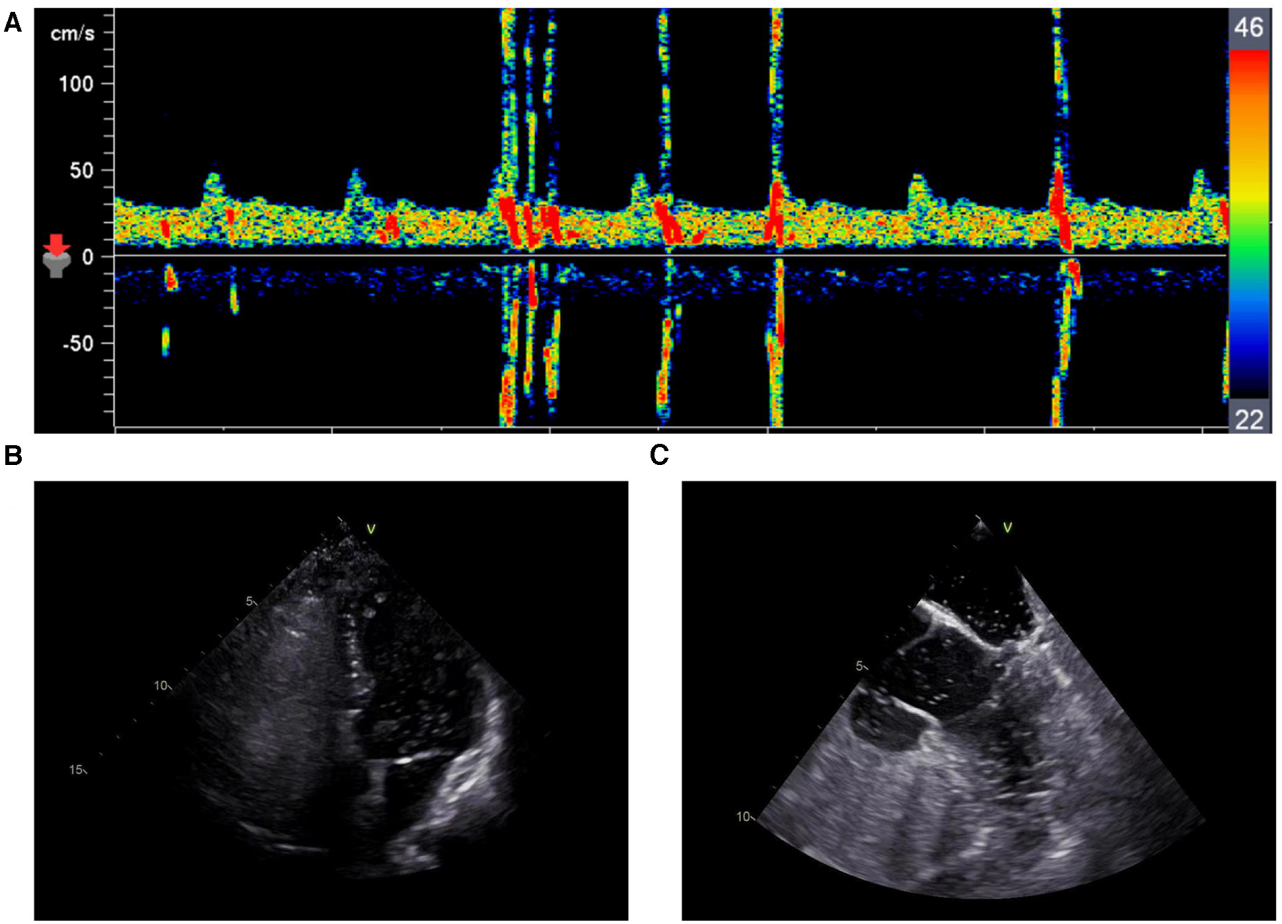
(**A**) c-TCD revealed a grade 2 right-to-left shunt during the valsalva maneuver. (**B**) c-TTE revealed a large number of microbubbles in the left-sided chambers during the Valsalva maneuver. (**C**) c-TEE revealed a large number of microbubbles in the left-sided chambers during the Valsalva maneuver.

This patient did not appear to be at a high risk for development of atherosclerosis; therefore, we attempted to determine whether there were cardio-embolic sources. Echocardiography revealed normal left ventricular ejection fraction without an intracardiac thrombus. Contrast-enhanced transcranial Doppler (c-TCD) revealed a grade 2 right-to-left shunt (24 microbubbles in the middle cerebral artery during the Valsalva maneuver) (Figure 3A). Contrast-enhanced transthoracic echocardiography (c-TTE) in this case revealed a moderate number of microbubbles in the left-sided chambers at rest and a large number of microbubbles during the Valsalva maneuver (Figure 3B). Transesophageal echocardiography (TEE) revealed a large (1.6 mm) long tunnel-shaped PFO (9.3 mm). Contrast-enhanced TEE (c-TEE) revealed a moderate-to-large number of microbubbles in the left atrium at rest and a large number of microbubbles during the Valsalva maneuver (Figure 3C).

**Figure 4:**
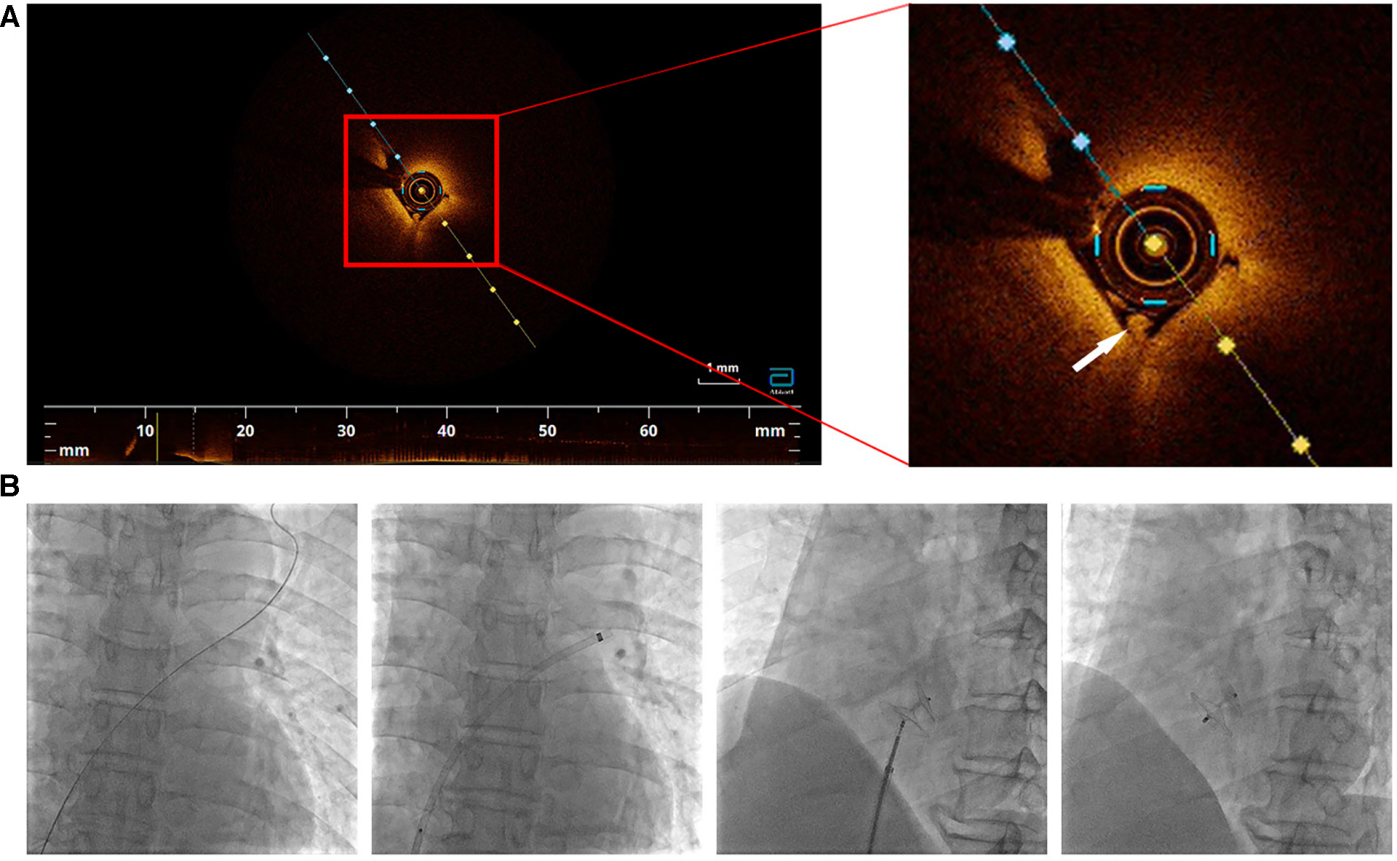
(**A**) Optical coherence tomography inside the tunnel of PFO. A white thrombus was observed (white arrow). (**B**) PFO closure was performed step by step.

We confirmed diagnosis of PFO based on these results, and the patient underwent percutaneous closure of the PFO. The right femoral vein was punctured under local anesthesia and a 0.014 inch balance middleweight coronary guidewire assisted by a 6F MPA2 catheter was used to probe through the PFO from the right to the left atrium and was subsequently advanced into the left superior pulmonary vein. After advancement of a 6F coronary guiding catheter along the guidewire into the right atrial entrance of the PFO, an optical coherence tomography (OCT) catheter (Abbott, USA) was used to observe the microstructure of the PFO. A white thrombus was observed in the tunnel (Figure 4A). We subsequently performed successful PFO occlusion using a 18/25 mm PFO occluder (Abbott, USA) (Figure 4B). The patient recovered well without any new cerebral infarction observed during 6-month follow-up.

## Discussion

The source of emboli remains unconfirmed in many patients with stroke. HES is classified into idiopathic, primary, and secondary HES; idiopathic HES is the most common type (4). HES may affect many organs, including the skin, lungs, gastrointestinal tract, and heart (2). This patient also had dermatological damage that manifested as localized erythema and erosion of the skin on the right lower extremity. However, no clinical evidence of cardiac or pulmonary complications were observed. Several studies have reported neurological impairment in patients with HES (6), and corticosteroids are administered as initial treatment in such cases (2). In this study, the patient received methylprednisolone, and his white blood cell count decreased to 8.47 × 10^9^/L with 1.4% eosinophils, 6 days after treatment.

Evidences have confirmed that HES is associated with multiple embolic events (7). A complete screening for thrombophilia in this patient, who presented cerebral infarction accompanied with HES, is mandatory. Laboratory tests such as antineutrophil cytoplasmic antibodies, anticardiolipin antibodies, antinuclear antibody profile, and common tumor markers were performed. No clinically meaningful abnormal results were found. Venous thrombi were also excluded by ultrasound of both lower limbs. However, computed tomography angiography (CTA) of the pulmonary artery and pelvic veins were not performed. This is a limitation that exists in our case. Screening for thrombophilia will be accomplished more thoroughly when we encounter similar cases in the future.

In patients with HES, stroke is invariably attributable to thromboembolism, which mainly occurs secondary to thrombi of cardiac origin (8). Moreover, in most cases, eosinophilia-associated cerebral infarction is not correlated with huge thromboembolism but with multiple infarcts in the watershed area. Microthromboemboli preferentially embolize along vessels to the border zones (7, 8). In our patient, transthoracic echocardiography (TTE) and TEE showed no evidence of atrial thrombus, and magnetic resonance imaging revealed cerebral infarction of the bilateral basal ganglia and corona radiata but not the watershed area.

PFO has received increasing attention in the last decade as an etiopathogenetic contributor to embolism and ischemic stroke. Previous studies have reported that PFO was associated with stroke in patients aged <60 years, particularly in those with cryptogenic stroke (9). Patients with PFO and right-to-left shunting at rest are considered to be at a high risk of stroke onset and recurrence (10).

However, paradoxical cerebral embolism secondary to PFO is invariably a presumptive diagnosis. Although TEE shows good sensitivity for detection of PFO, detection of thrombi in the PFO tunnel is challenging, and TEE-documented direct evidence of a thrombus lodged in the PFO is often unavailable (8). OCT is a novel imaging technique used to obtain high-resolution three-dimensional images of biological structures (11). OCT provides *in situ* and real-time images of tissue and is widely utilized for diagnostic purposes, with medical applications across several fields including ophthalmology, cardiology, otology, and dermatology (12). In this patient, we observed an OCT-documented thrombus lodged in the PFO.

In this study, we present direct imaging findings of an *in situ* thrombus in the PFO. Therefore, it is reasonable to presume PFO-associated development of paradoxical embolism. Based on the aforementioned results, PFO might be the etiopathogenetic contributor to stroke in our patient. Previous studies have reported proven clinical usefulness of PFO closure in prevention of recurrent stroke (13). However, prophylactic closure of PFO remains debatable. In this patient, we observed that PFO was the major cause of his cerebral infarction; therefore, PFO occlusion was a reasonable line of treatment.

To our knowledge, this report is the first to present OCT-documented direct imaging evidence of emboli that originated from the PFO in a patient with HES and concomitant PFO. Based on our findings in this study, we recommend that unusual etiologies of stroke should be considered, particularly in younger patients without classical cardiovascular risk factors.

## Data Availability

The original contributions presented in the study are included in the article/Supplementary Material, further inquiries can be directed to the corresponding authors.
